# Functional Connectivity of the Scene Processing Network at Rest Does Not Reliably Predict Human Behavior on Scene Processing Tasks

**DOI:** 10.1523/ENEURO.0375-24.2024

**Published:** 2025-02-11

**Authors:** David M. Watson, Timothy J. Andrews

**Affiliations:** Department of Psychology and York Neuroimaging Centre, University of York, York YO10 5DD, United Kingdom

**Keywords:** fMRI, functional connectivity, navigation, resting-state, spatial memory

## Abstract

The perception of scenes is associated with processing in a network of scene-selective regions in the human brain. Prior research has identified a posterior–anterior bias within this network. Posterior scene regions exhibit preferential connectivity with early visual and posterior parietal regions, indicating a role in representing egocentric visual features. In contrast, anterior scene regions demonstrate stronger connectivity with frontoparietal control and default mode networks, suggesting a role in mnemonic processing of locations. Despite these findings, evidence linking connectivity in these regions to cognitive scene processing remains limited. In this preregistered study, we obtained cognitive behavioral measures alongside resting-state fMRI data from a large-scale public dataset to investigate interindividual variation in scene processing abilities relative to the functional connectivity of the scene network. Our results revealed substantial individual differences in scene recognition, spatial memory, and navigational abilities. Resting-state functional connectivity reproduced the posterior–anterior bias within the scene network. However, contrary to our preregistered hypothesis, we did not observe any consistent associations between interindividual variation in this connectivity and behavioral performance. These findings highlight the need for further research to clarify the role of these connections in scene processing, potentially through assessments of functional connectivity during scene-relevant tasks or in naturalistic conditions.

## Significance Statement

Our ability to process scenes is crucial for interacting with our environment as it allows us to extract spatial, contextual, and navigational information. However, the mechanisms by which the scene network in the human brain supports these abilities remain poorly understood. To investigate this, we compared behavioral measures of scene processing with resting-state functional connectivity within the scene network. Extensive individual variability was evident in scene recognition, spatial memory, and navigational abilities. However, contrary to our preregistered hypothesis, we did not observe any consistent associations between task performance and the resting-state functional connectivity of the scene network. These results suggest that future research employing task-related or naturalistic designs may be necessary for elucidating the neural basis of scene perception.

## Introduction

The ability to extract spatial, contextual, and navigational information from visual scenes is key to how we interact with our environment. It is thought the processing of this information is underpinned by a core network of scene-selective regions in the human brain (Epstein and Baker, 2019). These include the parahippocampal place area (PPA; Epstein and Kanwisher, 1998) in the ventral temporal cortex, the retrosplenial complex/medial place area (RSC/MPA; Maguire, 2001; Silson et al., 2016) in the medial parietal cortex, and the occipital place area (OPA; Dilks et al., 2013) in the lateral occipital cortex. Additionally, an extended network of regions has also been implicated in scene processing. For instance, cortical patches directly anterior to the core scene regions have been linked to the representation and recall of familiar environments (Steel et al., 2021, 2023, 2024). Furthermore, medial temporal regions are implicated in spatial memory and navigation (O’Keefe and Nadel, 1978), while posterior parietal regions have been linked to visuospatial coding of scenes (Kravitz et al., 2011).

A full understanding of scene-selective regions requires an appreciation of how they interact with each other and wider brain networks. Previous studies measuring resting-state functional connectivity have proposed a bias between posterior and anterior aspects of the scene network (Baldassano et al., 2013, 2016; Nasr et al., 2013; Silson et al., 2016; Boccia et al., 2017a). The posterior network includes the OPA, and the anterior network includes the RSC, with both networks converging within the PPA. The posterior network connects more strongly with early visual regions and is thought to support processing of egocentric visual features. Meanwhile the anterior network is preferentially connected with more anterior extended scene regions and is thought to support higher-level mnemonic processing of scenes. Recently, Watson and Andrews (2024) demonstrated this posterior–anterior bias is also evident in functional connectivity measured during naturalistic viewing and in structural connectivity. They also found that posterior scene regions showed preferential connectivity with posterior parietal cortices, suggesting these regions may also support higher-level cognitive processes associated with the dorsal visual stream.

Resting-state functional connectivity is widely used to predict individual differences on cognitive tasks because it captures intrinsic brain network organization without task-specific demands. For example, individual variation in the resting-state functional connectivity of many brain networks has been shown to predict performance on numerous cognitive and behavioral measures (Stevens and Spreng, 2014; Finn et al., 2015; Rosenberg et al., 2016; Beaty et al., 2018; Ooi et al., 2022). Given that higher-level cognitive processes involved in processing scenes are thought to be supported by connections between the core scene network and other brain regions, a number of studies have investigated whether connectivity between specific regions is linked to performance on scene processing tasks. For instance, Sulpizio et al. (2016) demonstrated a positive correlation between performance on a self-report questionnaire of navigational ability and resting-state functional connectivity between the right RSC and posterior hippocampus. Other studies using task fMRI have found connectivity between the PPA, RSC, and hippocampus is modulated by perception and imagery of familiar places (Boccia et al., 2017b, 2019; Tullo et al., 2023). Furthermore, developmental topographic disorientation—a neurodevelopmental disorder marked by impaired navigational abilities (Iaria et al., 2009; Burles and Iaria, 2020)—has been associated with decreased functional connectivity between the RSC and PPA (Kim et al., 2015; but see Iaria et al., 2014). Nevertheless, previous research has largely focused on a limited number of behavioral tasks and connectivity among a relatively small set of brain regions. Consequently, there remains a gap in understanding how a broader selection of cognitive measures correlate with functional connectivity between scene regions and wider networks throughout the brain.

In this preregistered study, we utilize behavioral and resting-state fMRI data from a large-scale public dataset developed by Clark and Maguire (2023). This includes an extensive battery of cognitive behavioral measures, including tests of scene recognition, spatial memory, and navigational ability, in a large sample comprising over 200 participants. We used fMRI data to measure resting-state functional connectivity of the scene network in the same participants. We investigated the association between scene processing abilities and functional connectivity by comparing interindividual variation in functional connections to performance on the behavioral measures. We predicted that if connections subserve higher-level cognitive processing of scenes, then the strength of connectivity should be positively correlated with performance on behavioral measures of those processes. Contrary to our hypothesis, we did not find any consistent evidence of an association between human behavior and the functional connectivity of the scene network.

## Materials and Methods

This study was preregistered on the Open Science Framework (https://osf.io/m2qb6). 

### Dataset

We obtained behavioral and MRI data for 217 participants (109 females, 108 males, age range 20–41 years) from a publicly available dataset collected by Clark and Maguire (2023; https://doi.org/10.5061/dryad.2v6wwpzt3). This includes a comprehensive battery of cognitive behavioral measures assessing scene processing, memory, and navigational abilities. The dataset additionally includes resting-state fMRI data, which we used to measure functional connectivity. In brief, data were collected on 3 T Siemens MAGNETOM TIM Trio scanners. Whole-brain resting–state data were acquired in a single scan run lasting ∼13 min. High-resolution T1–weighted anatomical images were also acquired using a FLASH MRI sequence. We used the anatomical images acquired in the first scanning session, matching the session the resting-state data were acquired in. Full details are provided in Clark and Maguire (2023).

### Behavioral data analysis

We selected four cognitive behavioral measures that directly assess scene perception and navigational abilities:

1. We used the “scenes” subscale of the recognition memory (RM) test (Warrington, 1984; Cipolotti and Maguire, 2003), which tests participants' ability to learn and recognize previously unfamiliar images of buildings and landscapes.
2. The Santa Barbara Sense of Direction Scale (SBSOD) is a self-report questionnaire measuring spatial navigation ability (Hegarty, 2002). Participants rate 27 statements (e.g., “I am very good at judging distances”) for agreement on a Likert scale.
3. We used the “spatial” subscale of the Survey of Autobiographical Memory (SAM; Palombo et al., 2013), which provides a self-report questionnaire measuring spatial memory. Participants rate six statements (e.g., “In general, my ability to navigate is better than most of my family/friends”) for agreement using a Likert scale. The scores are then standardized, such that the expected population average is 100.
4. The navigation test (Woollett and Maguire, 2010) assesses real-world navigation ability. Participants watch videos depicting navigation along routes through an unfamiliar town and then are tested on their knowledge of the town's layout. We utilized data from all five subscales of this test: (1) clip recognition; (2) scene recognition; (3) proximity judgements between landmarks; (4) knowledge of the order of locations along the routes; and (5) the sketch map, in which participants draw the routes from memory. We also used the overall summary score, calculated by summing the scores over all subscales.

We reverse-scored values on the SBSOD, such that on all measures a higher score indicates better performance. To compare performance on the tasks, we conducted exploratory analyses correlating participants' scores between pairwise combinations of behavioral measures (including the overall score and all subscales for the navigation test). We derived two additional visualizations by converting the resulting correlations matrix into units of correlation distance. First, we applied hierarchical clustering, using an unweighted average distance linkage (implemented in *scipy*; Virtanen et al., 2020). Second, we applied metric multidimensional scaling (implemented in *scikit-learn*; Pedregosa et al., 2011).

### MRI data analysis

#### Preprocessing

The MRI data were preprocessed using FSL (Jenkinson et al., 2012). The T1 anatomical images were processed using the *fsl_anat* script provided with FSL (https://fsl.fmrib.ox.ac.uk/fsl/docs/#/structural/fsl_anat). First, a bias field correction is applied using FAST (Zhang et al., 2001). A nonlinear registration to the MNI152 standard space is then computed using FNIRT (Andersson et al., 2010). Removal of nonbrain structures is performed by transforming the MNI brain mask back to the T1 space. The brain extracted volume is then reprocessed with FAST to derive tissue segmentations. Finally, FIRST (Patenaude et al., 2011) is used to automatically segment subcortical structures.

The functional resting-state data were first preprocessed using FEAT. We removed three dummy volumes (10.08 s) from the start of the scan run and applied motion correction using MCFLIRT (Jenkinson et al., 2002), slice-timing correction, nonbrain removal using BET (Smith, 2002), spatial smoothing using a Gaussian kernel (FWHM, 6 mm; twice the voxel resolution), and grand-mean intensity normalization by a single multiplicative factor. Additionally, functional images were coregistered to the T1 anatomical images via boundary based registration (Greve and Fischl, 2009). Two additional denoising steps were then applied. First, MELODIC (Beckmann and Smith, 2004) derived spatiotemporal independent components from the data, and then ICA-AROMA (Pruim et al., 2015) automatically labeled noise components associated with head motion and regressed them out of the data. We employed an aggressive denoising strategy, such that all variance associated with noise components was removed from the data. High-pass temporal filtering was applied following the ICA denoising (σ = 50 s). Second, a component-based (CompCor) denoising approach (Behzadi et al., 2007) was applied to remove CSF-related signals. The CSF partial volume estimates derived from the tissue segmentations of the T1 images were transformed to the functional volumes and thresholded at 90%. The average time series and first four principal components from the CSF voxels were then regressed out of the data.

#### Regions of interest

We defined regions of interest (ROIs) for early visual, core scene, and extended scene areas in each hemisphere. The Clark and Maguire dataset does not contain a functional localizer, so we instead functionally defined the core scene regions from an independent fMRI dataset (Noad et al., 2024; https://openneuro.org/datasets/ds004848). In brief, 45 neurologically healthy subjects completed a functional localizer scan in which they viewed images of scenes, faces, and phase scrambled faces. A group-level analysis identified scene-selective activation using a contrast of “scenes > (faces + scrambled)”. We then defined ROIs for the OPA, PPA, and RSC. We applied a clustering algorithm to define clusters of 250 spatially contiguous voxels (2,000 mm^3^) around peak voxels in each region. Following previous studies (Baldassano et al., 2016; Silson et al., 2016; Watson and Andrews, 2024), we divided the PPA into posterior (pPPA) and anterior (aPPA) portions by splitting the full region along the *y*-axis to evenly balance the volumes of each subdivision (∼1,000 mm^3^ each). Each of these group-level ROIs was then transformed from the MNI space to the functional volumes of each participant in the Clark and Maguire dataset.

**Figure 1.**
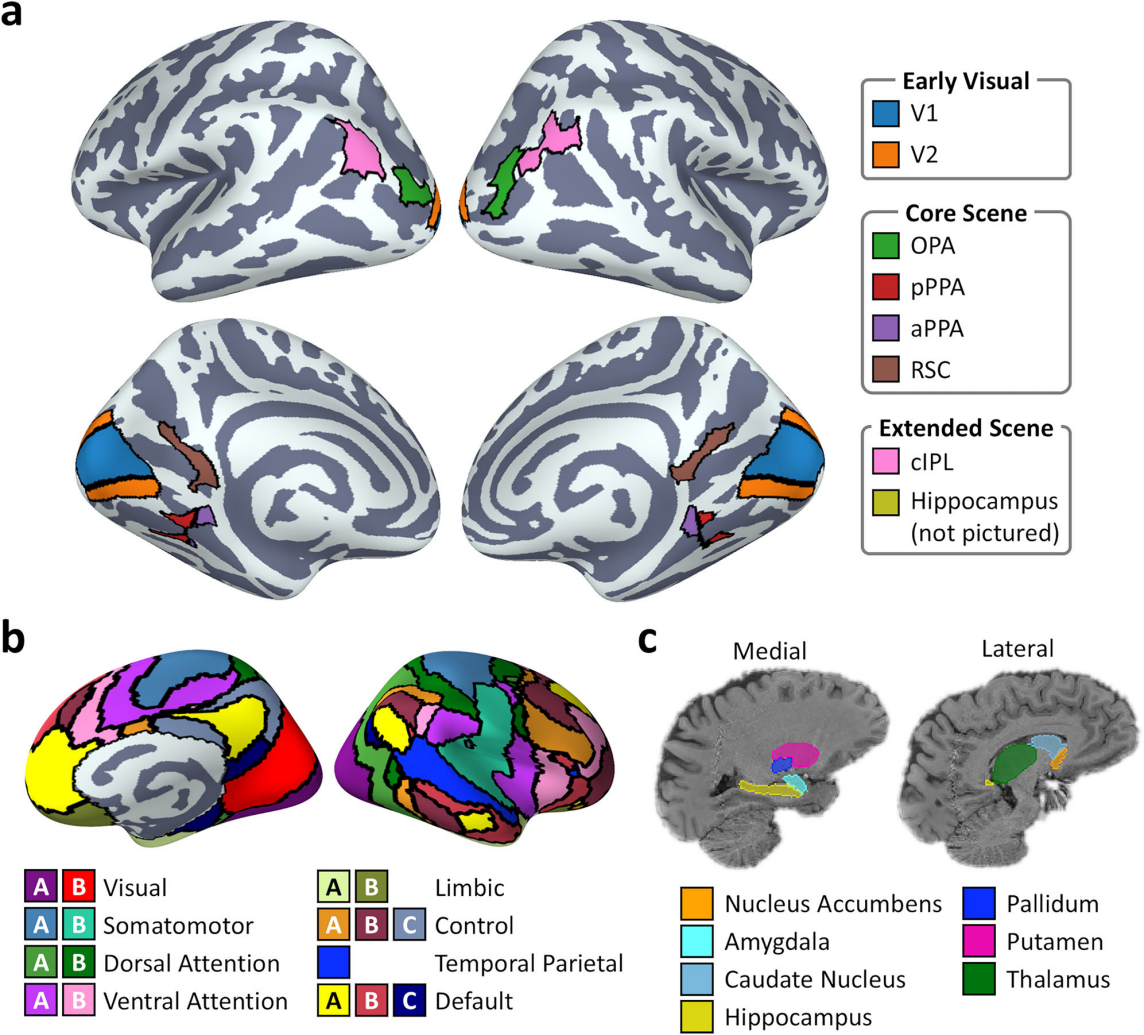
Locations of ROIs for connectivity analyses. ***a***, Main regions: early visual (V1, V2); core scene (OPA, pPPA, aPPA, RSC); extended scene [cIPL, hippocampus (not pictured)]. Note that surface projections of volumetric regions provide only approximate visualizations—see Extended Data Figure 1-1 for an alternative volume-based visualization. ***b***, 17 cortical resting-state networks from the Yeo atlas (Yeo et al., 2011). ***c***, Subcortical structures segmented from T1 anatomical scan.

Early visual ROIs for V1 and V2 were defined from the Wang retinotopic atlas (Wang et al., 2015). We additionally defined two extended scene regions that show preferential connectivity with the anterior core scene regions (Baldassano et al., 2016). We defined an ROI for the caudal inferior parietal lobule (cIPL) from the PGp region of the JuBrain/SPM Anatomy Toolbox (Eickhoff et al., 2005; Caspers et al., 2006). We also defined an ROI for the hippocampus using the subcortical segmentations of each participant's T1 scan. The locations of these ROIs are illustrated in Figure 1*a*.

10.1523/ENEURO.0375-24.2024.f1-1 Figure 1-1 Volume-based visualisation of main regions of interest for connectivity analyses: early visual (V1, V2); core scene (Occipital Place Area [OPA], posterior and anterior Parahippocampal Place Area [pPPA, aPPA], Retrosplenial Complex [RSC]); extended scene (caudal Inferior Parietal Lobule [cIPL], Hippocampus). Regions are displayed on the MNI152 brain. Download Figure 1-1, TIF file.

#### Functional connectivity

The preprocessed and denoised resting-state data were used to estimate functional connectivity. We first measured connectivity between the early visual (V1, V2), core scene (OPA, pPPA, aPPA, RSC), and extended scene regions (cIPL, hippocampus). Time series were averaged over voxels within each ROI and then correlated pairwise between regions. Correlations were converted to units of Fisher's *z*. This produced a correlations matrix for each participant representing the functional connectivity between regions. To visualize the overall pattern of connectivity, the matrices were averaged over participants then converted back to units of Pearson's *r*. Additional visualizations were derived by converting the group average matrix to units of correlation distance, then submitting this to both hierarchical clustering (using an average distance linkage) and metric multidimensional scaling.

We also examined the connectivity between the core scene regions (OPA, pPPA, aPPA, RSC) and the rest of the brain. We measured cortical connectivity between these regions and each of 17 resting-state networks across the brain (Yeo et al., 2011; Fig. 1*b*). To prevent double-dipping, we removed any voxels overlapping between a particular network and scene region from the network prior to calculating the connectivity. We additionally measured connectivity between each of the core scene regions and seven subcortical structures: the nucleus accumbens, amygdala, caudate nucleus, hippocampus, pallidum, putamen, and thalamus (Fig. 1*c*). The subcortical regions were identified from the automatic subcortical segmentation of the T1 scans. Note that the hippocampus region is the same as the one included in the extended scene ROIs.

For each of the connectivity analyses (main ROIs, core scene ROIs with Yeo networks, core scene ROIs with subcortical structures), we compared interindividual variation in functional connectivity with performance on the behavioral measures. The Fisher's *z* connectivity values for each connection across participants were correlated with scores on each behavioral measure. This produced an additional matrix for each behavioral measure, indicating the correlation between the behavioral performance and the functional connectivity for each connection. We applied a Holm–Bonferroni correction (Holm, 1979) for multiple comparisons over connections. We additionally calculated Bayes factors for each correlation, implemented with the *BayesFactor* R package (https://cran.r-project.org/package=BayesFactor) and using the default prior distribution.

To investigate whether behavior can be predicted from the interaction of multiple connections, we conducted exploratory analyses using a series of ridge regression models. For each connectivity analysis (main ROIs, core scene ROIs with Yeo networks, core scene ROIs with subcortical regions) and for each behavioral measure, we constructed a ridge regression model using all connections within the connectivity matrix as predictors and the behavioral measure as the outcome variable. Model performance was assessed using cross-validated *R*^2^ values, employing a five-fold cross-validation over participants. The regularization parameter for each model was optimized with a further five-fold cross-validation nested within each training set, using a Bayesian optimization routine to search over the parameter space (implemented with *scikit-optimize*; https://scikit-optimize.readthedocs.io).

Finally, we conducted seed-based analyses to provide a finer-grained examination of the connectivity between the core scene regions (OPA, pPPA, aPPA, RSC) and the rest of the brain. For each seed, the preprocessed and denoised time series were extracted and averaged over voxels within the region. This was then entered into a first-level FEAT analysis (Woolrich et al., 2001) as the sole regressor. Connectivity estimates for each seed were then combined over subjects in a series of higher-level mixed-effect FEAT analyses using FLAME (Woolrich et al., 2004). To estimate the overall pattern of connectivity, we conducted higher-level analyses calculating a single group average contrasting the connectivity against zero. To measure the correlation between connectivity and a particular behavioral measure, we extracted and demeaned the scores for that measure over participants. A design matrix for a higher-level analysis was then constructed including an intercept term plus a slope term comprising the demeaned scores. A cluster correction (Worsley, 2001) was applied to the statistical images, using a stringent cluster forming threshold of *Z* > 4.9 (two-tailed *p* < 10^−6^) for the group average and a laxer threshold of *Z* > 3.3 (two-tailed *p* < 0.001) for the behavioral analyses and a cluster significance threshold of *p* < 0.05.

### Deviations from preregistration

We note the following deviations from our preregistered design plan. First, we proposed to use all behavioral measures available in the Clark and Maguire dataset and apply exploratory factor analysis to identify a smaller number of factors for comparison with the functional connectivity. However, the behavioral measures cover a very wide range of cognitive abilities beyond just scene processing, and consequently the resulting factors did not clearly identify scene perception or navigational abilities. We therefore opted to manually select the most relevant behavioral measures instead. Second, the correlation analyses between behavioral measures and the ridge regression analyses predicting behavior from functional connectivity were not preregistered.

## Results

### Behavioral data analysis

We obtained behavioral data from four measures of scene processing and navigational ability included in the Clark and Maguire (2023) dataset. The RM test measures the ability to learn and remember novel scenes. The SBSOD and spatial subscale of the SAM measure self-reported spatial navigation and memory abilities. The navigation test measures real-world scene processing abilities by having participants learn novel routes around an unfamiliar environment. This includes subscales measuring clip and scene recognition, proximity judgments between landmarks, knowledge of locations along the routes, and a sketch map reproducing the layout of the environment, plus an overall score combining all subscales.

**Figure 2.**
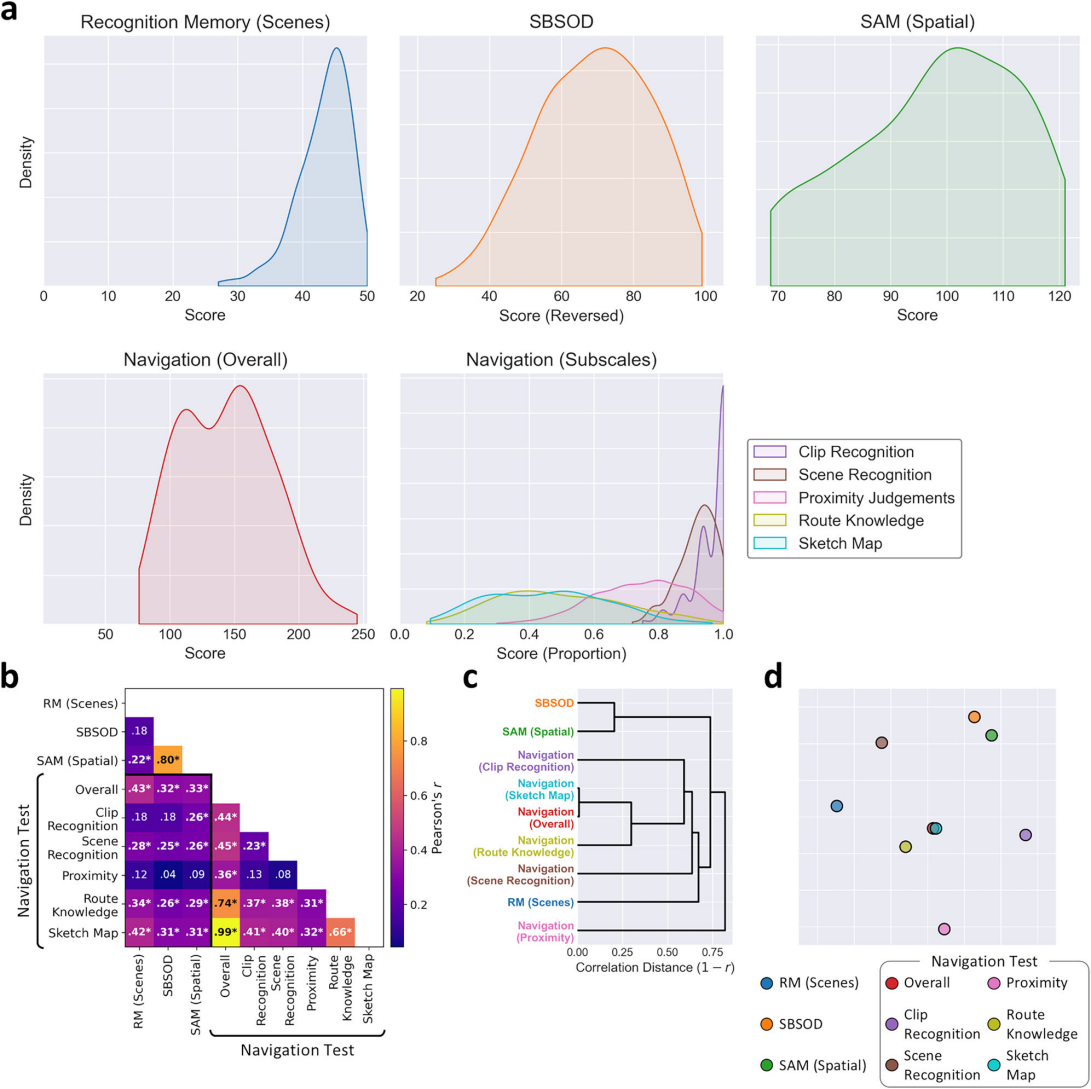
Behavioral measures of scene processing: RM for scenes; SBSOD; SAM (spatial subscale); navigation test (overall score and subscales). ***a***, Kernel density estimates illustrating distributions of scores on each measure. Higher scores indicate better performance. The horizontal axes extend to the limits of each measure, except the SAM where the axis extends to the data limits. ***b***, Correlations between measures. Significant correlations are labeled in bold with an asterisk (*p* < 0.05; FWER corrected). ***c***, Hierarchical clustering and (***d***) multidimensional scaling visualizations of the correlation matrix.

We first conducted exploratory analyses of the behavioral performance on each measure. Figure 2*a* shows kernel density estimates illustrating the distribution of scores across participants for each measure. There was a slight ceiling effect for RM, while the SBSOD, spatial SAM, and overall score on the navigation test all indicated broad distributions of scores. Among the subscales of the navigation test, the clip and scene recognition measures showed slight ceiling effects, while the proximity judgments, route knowledge, and sketch map showed broader distributions. Thus, a wide range of scene processing abilities were observed across participants.

To compare the different tasks, we correlated the scores from individual participants for all pairwise combinations of measures (Fig. 2*b*). Alternative visualizations are also provided by hierarchical clustering (Fig. 2*c*) and multidimensional scaling (Fig. 2*d*). This highlighted a wide range of correlations between measures, from *r* = 0.04 (between the SBSOD and proximity judgments subscale of the navigation test) up to *r* = 0.99 (between the sketch map subscale and overall score of the navigation test). This indicates that different measures were able to assess different aspects of scene processing. The overall score and sketch map subscale of the navigation test had the highest correlation—the sketch map has the highest maximum score of all the subscales and hence makes the largest contribution to the sum over all scores. High correlations were also observed between the route knowledge subscale and both the overall score (*r* = 0.74) and sketch map subscale (*r* = 0.66) of the navigation test. A strong correlation was also observed between the SBSOD and spatial SAM (*r* = 0.80). However, the proximity judgments subscale of the navigation test showed relatively weaker correlations with the other measures and indeed appeared more distant in the hierarchical clustering and multidimensional scaling visualizations, indicating this may reflect somewhat different aspects of scene processing to the other measures.

In summary, each of the behavioral measures captured wide individual differences in scene processing abilities. Correlations between measures highlighted a wide range of correlations, with performance appearing highly consistent between some tasks and more distinct between others. This indicates that different tasks assessed different aspects of cognitive processing of scenes.

### Functional connectivity of scene regions

**Figure 3.**
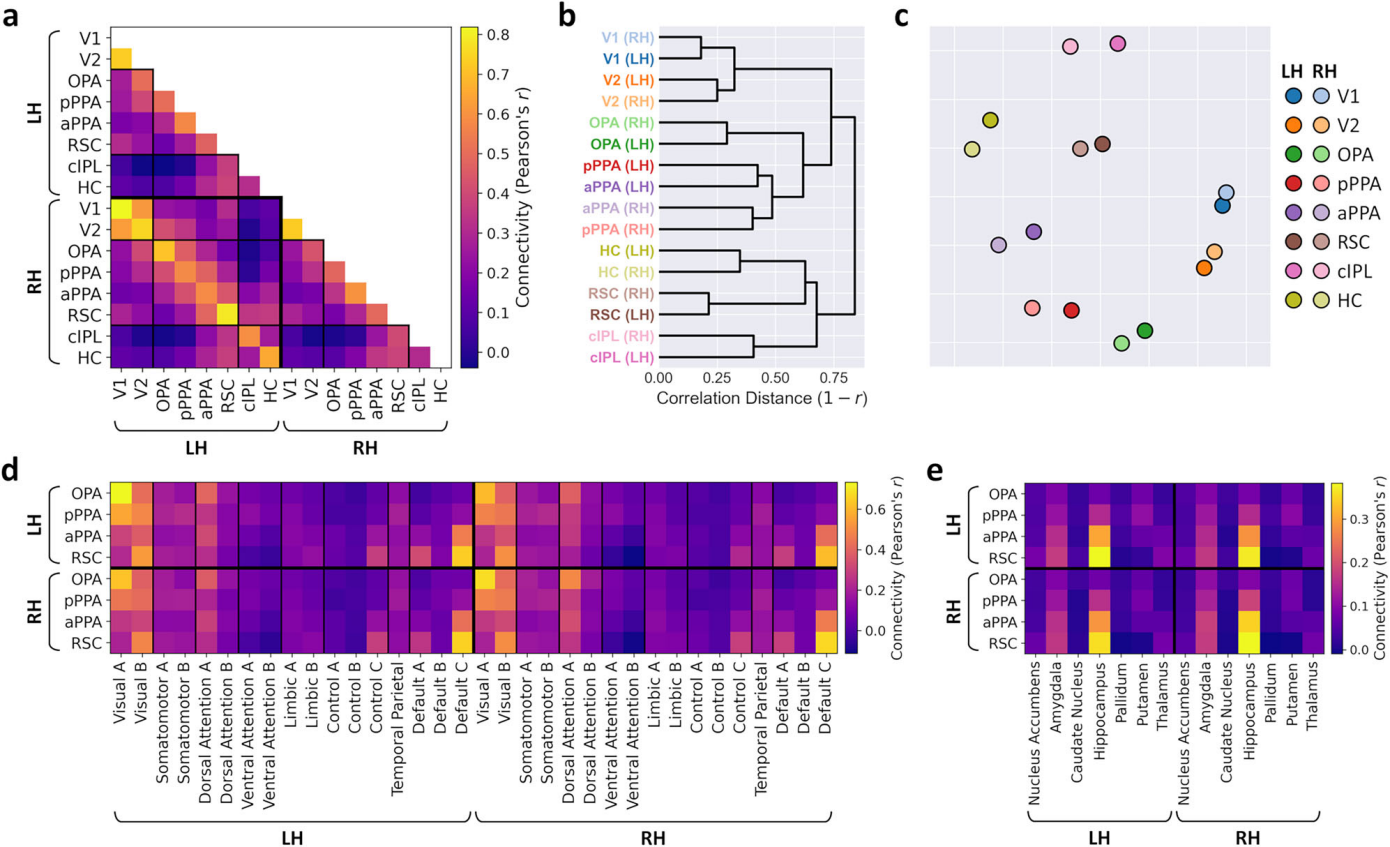
Group average functional connectivity between ROIs. ***a***, Connectivity between early visual, core scene, and extended scene regions. ***b***, Hierarchical clustering and (***c***) multidimensional scaling representations of the connectivity matrix. Connectivity of the core scene regions was also measured with (***d***) 17 cortical resting-state networks and (***e***) subcortical regions. See Extended Data Figure 3-1 for split-half reliability estimates of the functional connectivity.

We next measured the resting-state functional connectivity of the scene network. We first estimated the functional connectivity between early visual (V1 and V2), core scene (OPA, pPPA, aPPA, RSC), and extended scene (cIPL, hippocampus) ROIs (Fig. 1*a*). The group average connectivity matrix between these regions is illustrated in Figure 3*a*. Alternative visualizations are also provided by hierarchical clustering (Fig. 3*b*) and multidimensional scaling (Fig. 3*c*). Consistent with previous research, we observed a bias between more posterior scene regions (OPA and pPPA), which connected more strongly with the early visual regions, and more anterior scene regions (aPPA and RSC), which connected more strongly with the extended scene regions.

10.1523/ENEURO.0375-24.2024.f3-1 Figure 3-1 Split-half reliability of functional connectivity between first and second halves of resting-state scan runs. Matrices illustrate group average functional connectivity between (a) main regions of interest, (b) core scene regions and 17 cortical resting-state networks, and (c) core scene regions and subcortical regions for each data split. (d) Split-half correlations between connectivity matrices. Violin plots illustrate distributions of correlations over individual subjects. Diamond markers indicate correlations between group average matrices. Download Figure 3-1, TIF file.

We next conducted a series of analyses estimating functional connectivity between the core scene regions and the rest of the brain. We first measured connectivity with 17 cortical resting-state networks (Yeo et al., 2011) throughout the brain (Figs. 1*b*, 3*d*). We observed preferential connectivity between posterior scene regions (OPA, pPPA) and Component A of the visual network, which includes posterior occipital regions. The OPA also displayed preferential connectivity with Component A of the dorsal attention network, spanning lateral occipital and posterior parietal cortices. By comparison, anterior scene regions (aPPA, RSC) indicated stronger connectivity with Component C of the frontoparietal control network and Components A and C of the default mode network. These networks include medial and lateral parietal, dorsolateral and ventromedial prefrontal, and medial temporal cortices.

We also measured connectivity between the core scene regions and seven subcortical structures (Figs. 1*b*, 3*e*). Duplicating the analysis from the main ROIs (compare Fig. 3*a*), preferential connectivity was observed between anterior scene regions and the hippocampus. Anterior scene regions also showed stronger connectivity with the amygdala and to some extent the thalamus. The PPA and OPA also displayed moderate connectivity with the putamen.

To determine the reliability of these connectivity patterns, we split the resting-state data between the first and second halves of the scan runs and repeated the above connectivity analyses for each split. Extended Data Figure 3-1 indicates that connectivity patterns were stable across the splits, and high split-half correlations were observed between the connectivity matrices across subjects for all analyses.

**Figure 4.**
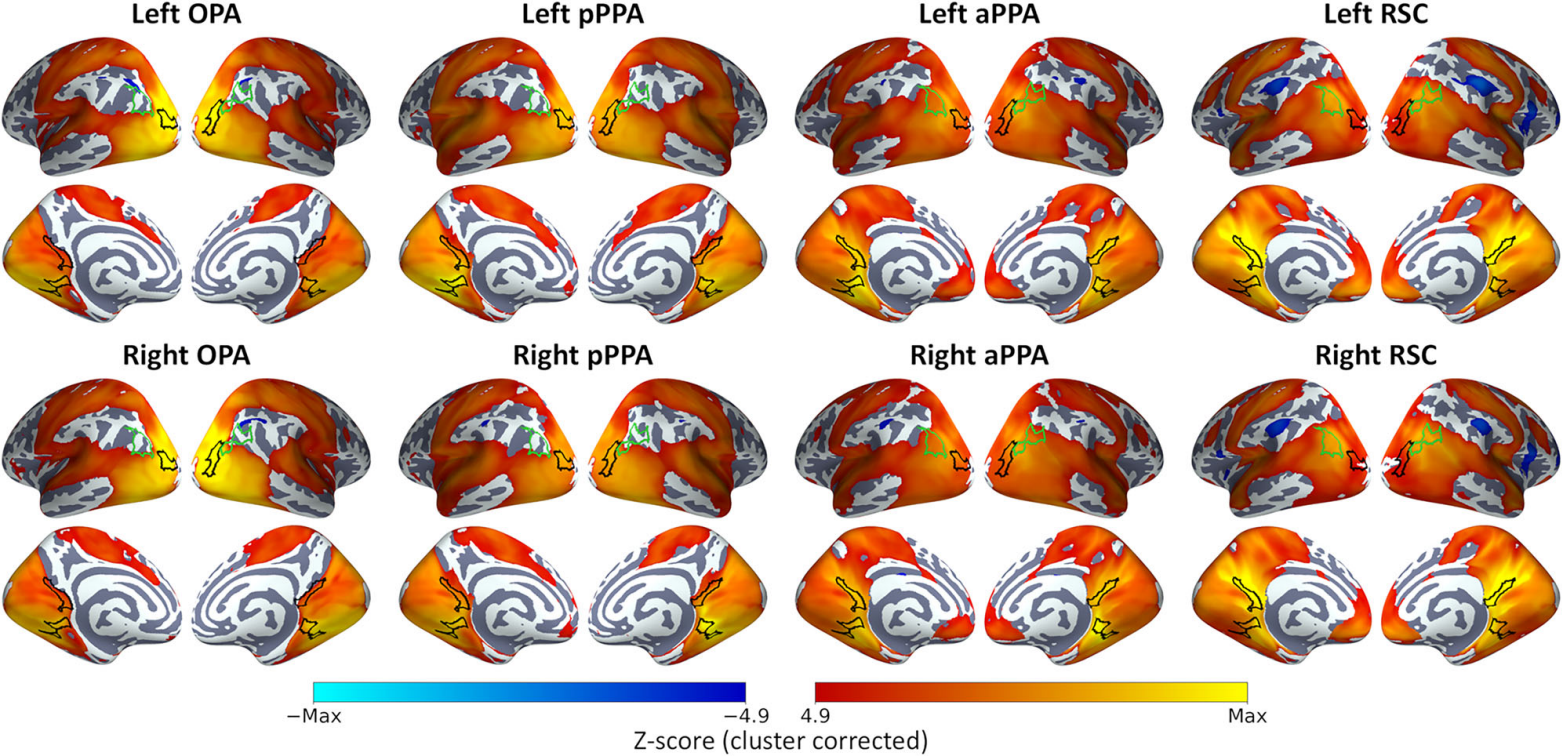
Group average seed-based functional connectivity for each core scene region. Statistical overlays illustrate cluster-corrected *Z*-scores for one-sample tests of connectivity against zero. Black outlines indicate locations of core scene regions (OPA, PPA, RSC), and the green outline indicates the location of the cIPL.

Finally, to provide a finer-grained measure of whole-brain connectivity, we conducted a series of seed-based analyses measuring the connectivity between the core scene regions and all voxels throughout the brain. Figure 4 shows group-level statistical maps for each seed region. All of the core scene regions highlighted extensive bilateral functional connectivity, particularly throughout occipital and inferior temporal cortices, but also extending into parietal and frontal cortices.

In summary, the resting-state analyses replicated previous results (Baldassano et al., 2016; Silson et al., 2016; Watson and Andrews, 2024) indicating extensive bilateral functional connectivity of the core scene regions and connectivity biases between the posterior and anterior regions. We next sought to compare interindividual variability in this functional connectivity with behavioral performance on measures of scene perception.

### Functional connectivity and behavior

**Figure 5.**
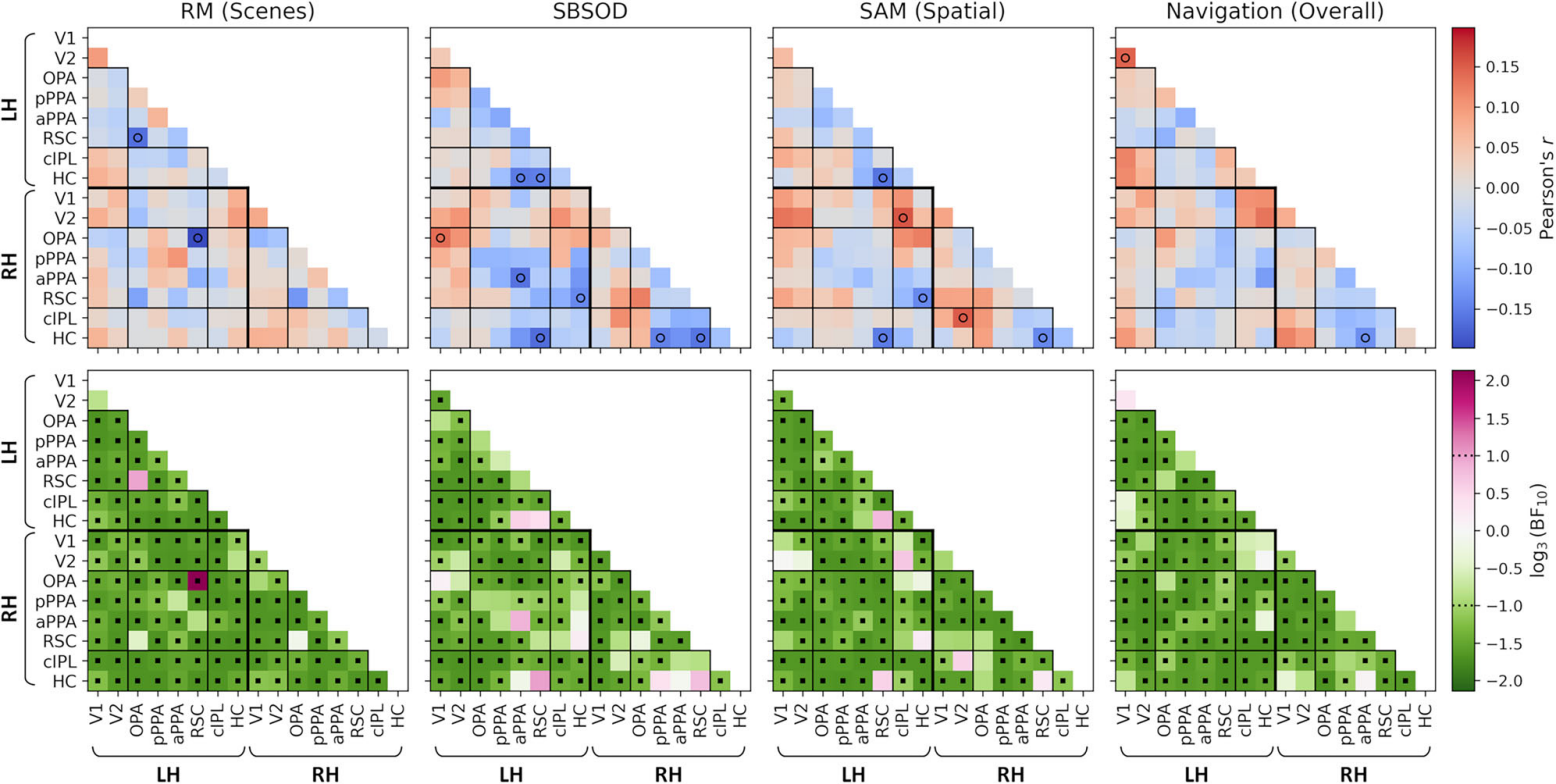
Correlations between behavioral measures (RM for scenes; SBSOD; SAM, spatial subscale; navigation test, overall score) and functional connectivity between early visual, core scene, and extended scene regions (compare Fig. 3*a*). The top row illustrates correlation values—open circles indicate correlations significant at an uncorrected level (*p* < 0.05). No correlations survived a FWER correction. The bottom row illustrates Bayes factors on a log scale—positive and negative values indicate support for the alternative and null hypotheses, respectively. Square markers indicate Bayes factors >3 or <1/3. Correlations with subscales of the Navigation test are illustrated in Extended Data Figure 5-1.

We first compared functional connectivity between the main ROIs (early visual, core scene, and extended scene; compare Fig. 3*a*) with human behavior. For each connection in the connectivity matrix, the correlation values were themselves correlated with the scores on each behavioral measure. Figure 5 illustrates results for the main behavioral measures—RM for scenes, SBSOD, spatial subscale of the SAM, and the overall score on the real-world navigation test. Contrary to our hypothesis, we did not observe any consistent relationships between any of the functional connections and behavioral measures. The resulting correlations included both positive and negative effects and indicated only small effect sizes (approximately in the range *r* = ±0.2). A small number of the correlations were significant; however, none of these survived a correction for multiple comparisons. The corresponding Bayes factors indicated moderate-to-strong support for the null hypothesis in the majority of cases. Analysis of the subscales of the navigation test revealed a similar pattern of results (Extended Data Fig. 5-1).

10.1523/ENEURO.0375-24.2024.f5-1 Figure 5-1 Correlations between subscales of the Navigation test and functional connectivity between early visual, core scene, and extended scene regions (cf. Figure 2a). Top row illustrates correlation values – open circles indicate correlations significant at an uncorrected level (p < 0.05). No correlations survived the FWER correction. Bottom row illustrates Bayes factors on a log scale – positive and negative values indicate support for the alternative and null hypotheses respectively. Square markers indicate Bayes factors greater than 3 or less than 1/3. Download Figure 5-1, TIF file.

**Figure 6.**
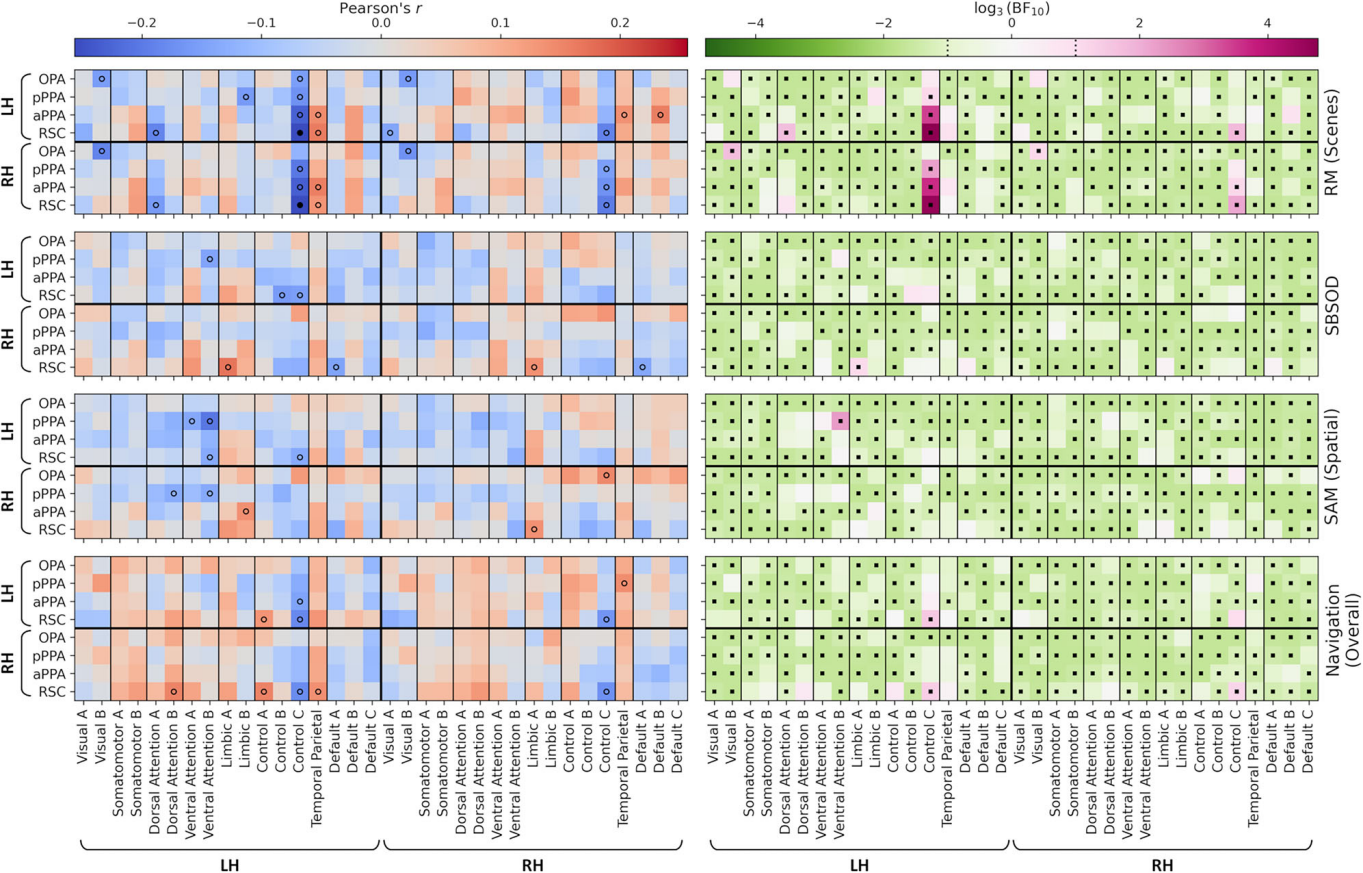
Correlations between behavioral measures (RM for scenes; SBSOD; SAM, spatial subscale; navigation test, overall score) and functional connectivity between core scene regions and 17 cortical resting-state networks (compare Fig. 3*d*). The left column illustrates correlation values—open circles indicate correlations significant at an uncorrected level; filled circles indicate correlations significant following a FWER correction (*p* < 0.05). The right column illustrates Bayes factors on a log scale—positive and negative values indicate support for the alternative and null hypotheses, respectively. Square markers indicate Bayes factors >3 or <1/3. Correlations with subscales of the navigation test are illustrated in Extended Data Figure 6-1.

Associations with behavior may be better evident in the functional connectivity measured between the scene network and other higher-level networks through the rest of the brain. To this end, we next considered the functional connectivity measured between the core scene regions and cortical resting-state networks (Yeo et al., 2011; compare Fig. 3*d*). Correlations for the main behavioral measures are illustrated in Figure 6 and for the navigation test subscales in Extended Data Figure 6-1. However, this again yielded only small effects and included both positive and negative correlations. Two significant negative correlations, surviving the FWER correction, were observed for the comparison of RM to the connections between the right frontoparietal control Network C and both the left RSC (*r*_(215)_ = −0.26; *p* = 0.037; BF_10_ = 191.97) and right RSC (*r*_(215)_ = −0.25; *p* = 0.046; BF_10_ = 156.90). A small number of other correlations were significant but did not survive the FWER correction. Bayes factors indicated support for the null hypothesis in the majority of cases. Thus, there were no consistent relationships between behavioral performance and connectivity of the scene regions to other cortical networks throughout the brain.

10.1523/ENEURO.0375-24.2024.f6-1 Figure 6-1 Correlations between subscales of the Navigation test and functional connectivity between core scene regions and cortical resting-state networks (cf. Figure 2d). Left column illustrates correlation values – open circles indicate correlations significant at an uncorrected level (p < 0.05). No correlations survived the FWER correction. Right column illustrates Bayes factors on a log scale – positive and negative values indicate support for the alternative and null hypotheses respectively. Square markers indicate Bayes factors greater than 3 or less than 1/3. Download Figure 6-1, TIF file.

**Figure 7.**
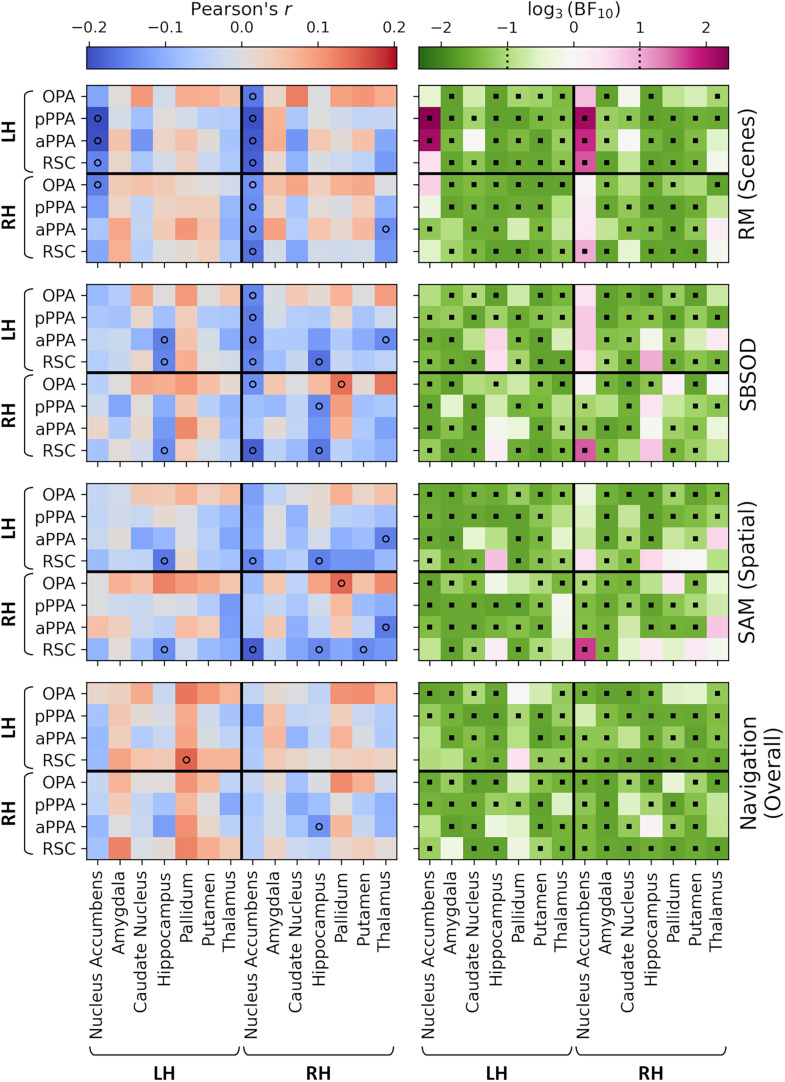
Correlations between behavioral measures (RM for scenes; SBSOD; SAM, spatial subscale; navigation test, overall score) and functional connectivity between core scene and subcortical regions (compare Fig. 3*e*). The left column illustrates correlation values—open circles indicate correlations significant at an uncorrected level (*p *< 0.05). No correlations survived the FWER correction. The right column illustrates Bayes factors on a log scale—positive and negative values indicate support for the alternative and null hypotheses, respectively. Square markers indicate Bayes factors >3 or <1/3. Correlations with subscales of the navigation test are illustrated in Extended Data Figure 7-1.

We next applied the same approach to the functional connectivity between core scene regions and subcortical structures (compare Fig. 3*e*). Correlations for the main behavioral measures are illustrated in Figure 7 and for the navigation test subscales in Extended Data Figure 7-1. Again, these analyses indicated a mix of small positive and negative correlations. The comparison of the clip recognition subscale of the navigation test with connectivity between the left OPA and left putamen revealed a positive correlation that was significant after correction for multiple comparisons (*r*_(215)_ = 0.26; *p* = 0.014; BF_(10)_ = 211.45). No other comparisons survived the FWER correction, and Bayes factors indicated support for the null hypothesis in the majority of cases. Therefore, once again, connectivity between scene and subcortical regions did not indicate any consistent relationship with behavior.

10.1523/ENEURO.0375-24.2024.f7-1 Figure 7-1 Correlations between subscales of the Navigation test and functional connectivity between core scene and subcortical regions (cf. Figure 2e). Left column illustrates correlation values – open circles indicate correlations significant at an uncorrected level; filled circles indicate correlations significant following a FWER correction (p < 0.05). Right column illustrates Bayes factors on a log scale – positive and negative values indicate support for the alternative and null hypotheses respectively. Square markers indicate Bayes factors greater than 3 or less than 1/3. Download Figure 7-1, TIF file.

**Figure 8.**
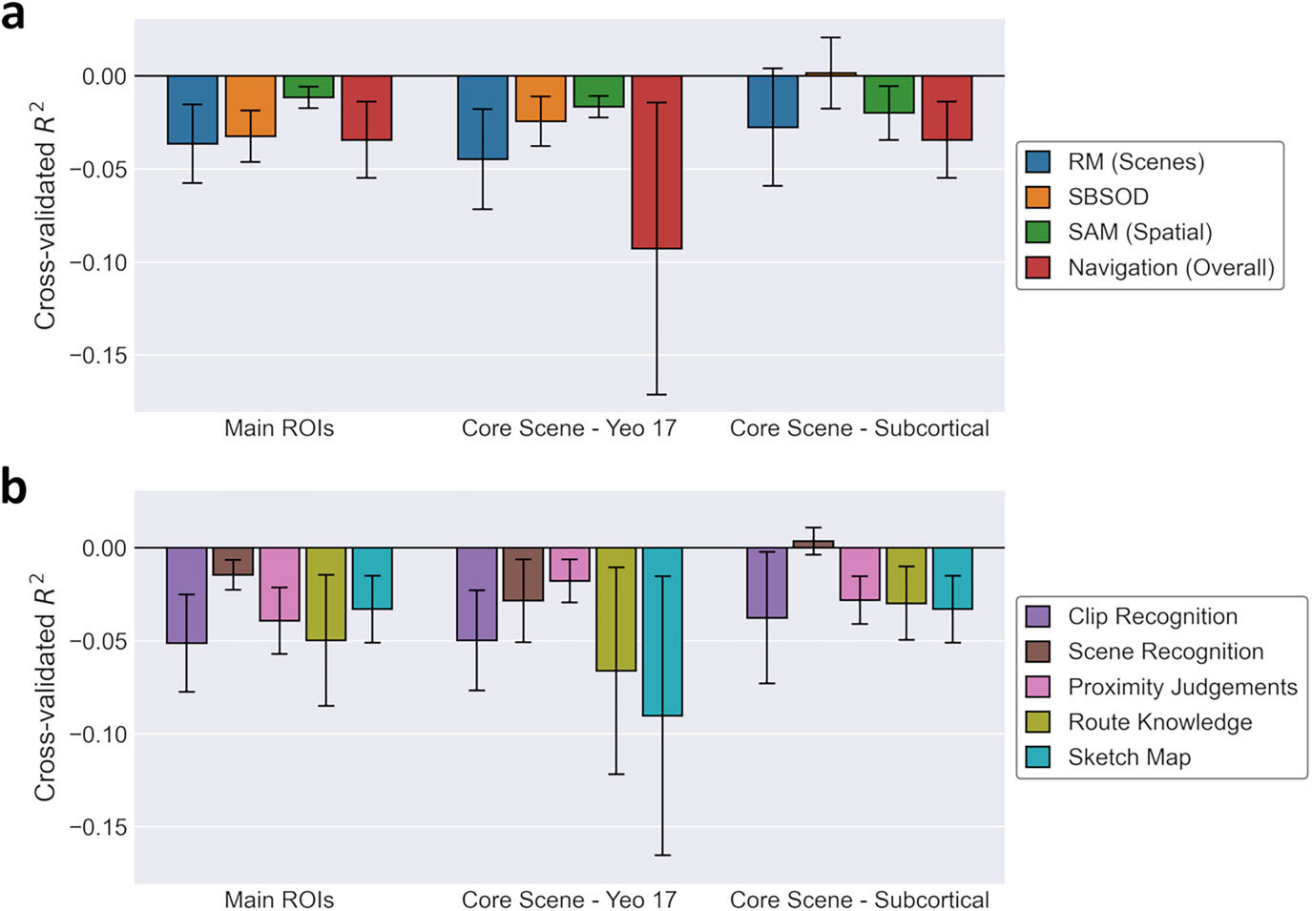
Results of ridge regression analyses, predicting behavioral measures from functional connectivity across all connections between: main ROIs (early visual, core scene, extended scene; compare Fig. 3*a*), core scene regions and cortical resting-state networks (compare Fig. 3*d*) and core scene and subcortical regions (compare Fig. 3*e*). Plots illustrate mean and standard error of model *R*^2^ values over cross-validation folds. ***a***, Prediction of main behavioral measures (RM for scenes; SBSOD; SAM, spatial subscale; navigation test, overall score). ***b***, Prediction of navigation test subscales.

The above analyses all considered the correlations between behavior and functional connectivity for each connection independently. It is possible that behavior could be better predicted from the interaction among multiple connections. To this end, we conducted exploratory analyses employing ridge regression models using all connections in each connectivity matrix (main ROIs, core scene—Yeo networks, core scene—subcortical) to predict scores on each behavioral measure. These models were cross-validated across participants, such that model performance was assessed via the coefficient of determination (*R*^2^) for participants held out from the model fitting. The resulting *R*^2^ values were small and often negative for all behavioral measures (Fig. 8), indicating that the regression models typically explained even less variance than a simple mean intercept model in the held-out participants. Thus, there was no indication that the interaction among connections can predict behavioral performance accurately.

**Figure 9.**
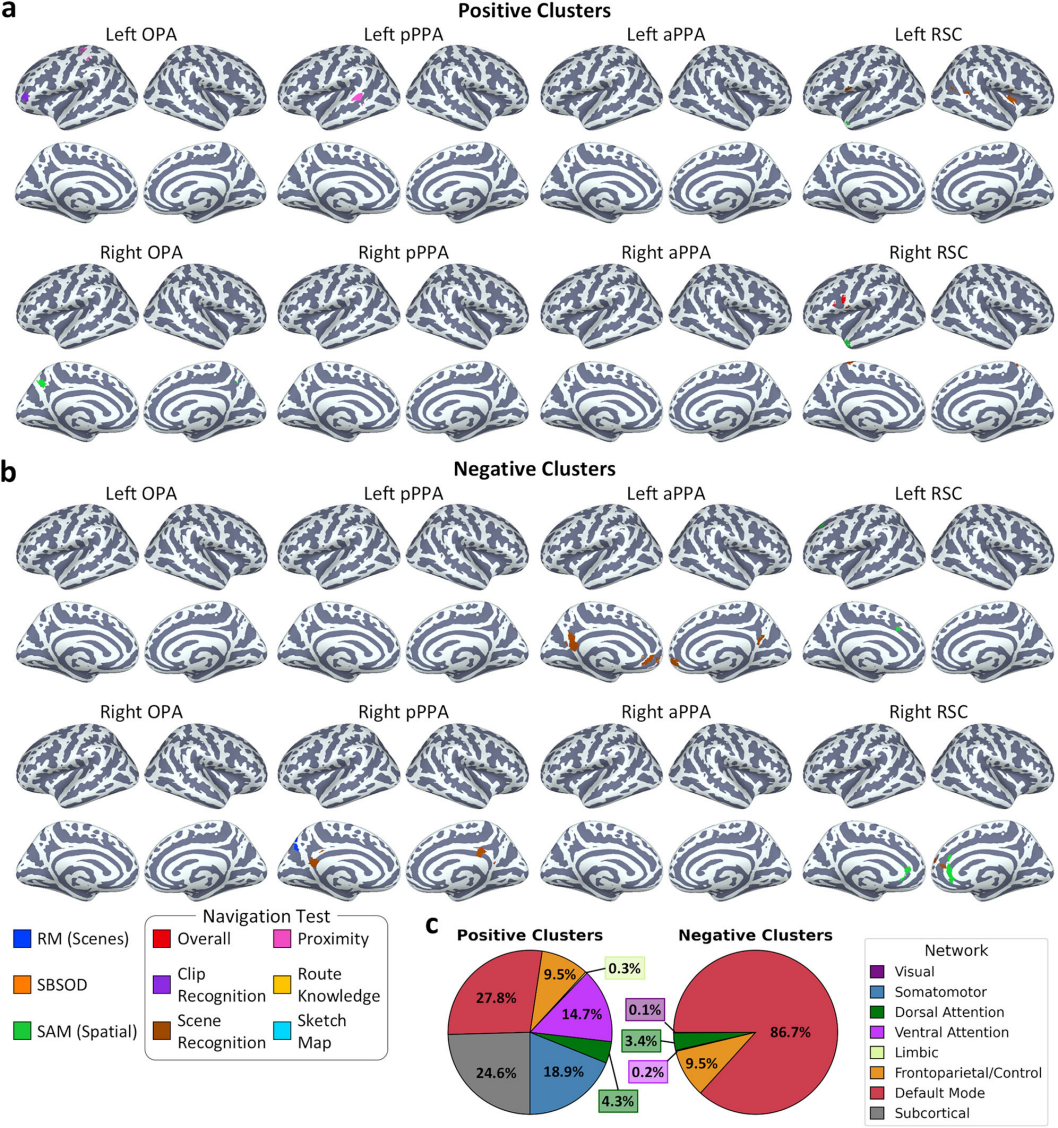
Correlations between behavior and seed-based functional connectivity. Brain plots illustrate clusters showing (***a***) positive and (***b***) negative correlations between connectivity and behavior for each seed region (*Z* > 3.3; two-tailed *p* < 0.001). ***c***, The percentage of voxels across all clusters overlapping with seven cortical resting-state networks from the Yeo atlas (Yeo et al., 2011) and subcortical structures.

Finally, we compared behavioral scores to the seed connectivity analyses to test whole-brain effects at a finer scale. Group-level analyses were conducted for each seed and for each behavioral measure, including the behavioral scores as a slope parameter. We identified voxel clusters showing significant (*Z* > 3.3; two-tailed *p* < 0.001) positive or negative associations with the behavioral scores following a cluster correction. We observed a number of relatively small clusters showing positive correlations with performance on the spatial SAM and scales of the navigation test, but not the SBSOD or RM tests (Table 1; Fig. 9*a*). To quantify the locations of the clusters, we calculated the percentage of voxels within the union of all clusters that overlapped subcortical structures and each of seven cortical resting-state networks (Yeo et al., 2011; Fig. 9*c*). The clusters did not consistently overlap any particular networks and instead were spread across multiple networks and subcortical structures in varying proportions (although no voxels overlapped the visual network). We also identified a number of relatively small clusters showing negative correlations with performance on the RM, spatial SAM, and navigation tests, but not the SBSOD (Table 2; Fig. 9*b*). The clusters were primarily located within the default mode network (Fig. 9*c*). No voxels overlapped the somatomotor or limbic networks or any subcortical structures, and the remaining voxels were distributed among other networks in smaller proportions. Thus, the seed-based analyses revealed only relatively small clusters, which were not reliably observed across behavioral measures. Furthermore, the positively correlated clusters did not consistently associate with the dorsal attention, frontoparietal control, or default mode networks which the scene regions show connectivity biases for.

**Table 1.** Locations of voxel clusters showing significant positive correlations between seed connectivity and behavioral measures

Seed	Measure	Cluster size (mm^3^)	Peak MNI coordinate (*x*, *y*, *z*; mm)	Cluster region
Left OPA	Navigation (clip recognition)	2,632	−26, 12, 2	Left putamen
		1,880	26, 12, 2	Right putamen
		944	−44, 42, 4	Left frontal pole
	Navigation (proximity judgments)	896	−36, −32, 52	Left postcentral gyrus
Right OPA	SAM (spatial)	1,856	−4, −60, 42	Left precuneus
Left pPPA	Navigation (proximity judgments)	1,256	10, −62, −26	Bilateral cerebellum
		1,192	−58, −40, 6	Left superior temporal sulcus
Left RSC	SAM (spatial)	984	−54, 14, −26	Left temporal pole
	Navigation (route knowledge)	1,120	18, −82, −44	Right cerebellum
	Navigation (scene recognition)	1,296	−62, 8, 4	Left precentral gyrus
		1,224	38, 2, 6	Right insula
		808	44, −50, 10	Right middle temporal gyrus
Right RSC	SAM (spatial)	2,000	−50, 16, −24	Left temporal pole
	Navigation (overall)	880	−50, 10, 24	Left inferior frontal gyrus
	Navigation (scene recognition)	1,736	−6, −36, 70	Left postcentral gyrus
		784	−62, 10, 6	Left precentral gyrus

**Table 2.** Locations of voxel clusters showing significant negative correlations between seed connectivity and behavioral measures

Seed	Measure	Cluster size (mm^3^)	Peak MNI coordinate (x, y, z; mm)	Cluster region
Right pPPA	RM (scenes)	1,424	0, −74, 52	Left precuneus
	Navigation (scene recognition)	992	−4, −48, 18	Left posterior cingulate
		888	4, −36, 34	Right posterior cingulate
Left aPPA	Navigation (scene recognition)	542	10, 52, −6	Bilateral ventromedial prefrontal cortex
		326	−6, −52, 16	Bilateral posterior cingulate
Left RSC	SAM (spatial)	928	−16, 26, 36	Left cingulate sulcus
Right RSC	SAM (spatial)	2,448	4, 32, 2	Bilateral anterior cingulate
	Navigation (scene recognition)	912	10, 54, 20	Right superior frontal gyrus

In summary, ROI-based analyses did not consistently identify any associations between resting-state functional connectivity of the scene network and behavioral measures of scene processing. Instead, the results revealed only weak effects, including both positive and negative correlations, and corresponding Bayes factors indicated support for the null hypothesis in the majority of cases. Seed-based analyses identified a number of clusters throughout the brain showing both positive and negative correlations between functional connectivity and behavior. However, these clusters were relatively small and were not observed reliably over the behavioral measures. Furthermore, the positively correlated clusters did not consistently identify any particular brain networks, although negatively correlated clusters did frequently overlap the default mode network.

## Discussion

In this study, we utilized data from a large-scale public dataset (Clark and Maguire, 2023) to investigate the relationship between resting-state functional connectivity of the scene network and performance on cognitive behavioral measures of scene processing. Our analysis revealed substantial individual differences in scene recognition, spatial memory, and navigational abilities. We also observed a wide range of correlations between measures, indicating consistent performance across some tasks but greater divergence between others. However, contrary to our preregistered hypothesis, we did not find any consistent relationship between the resting-state functional connectivity of the scene network and behavioral performance.

We first conducted exploratory analyses of behavioral measures related to scene processing ability. These included an RM test for scenes (Warrington, 1984; Cipolotti and Maguire, 2003); the SBSOD measuring self-reported navigation ability (Hegarty, 2002); a subscale of the SAM assessing self-reported spatial memory (Palombo et al., 2013); and a navigation test measuring real-world route learning and navigational ability (Woollett and Maguire, 2010). We observed extensive individual differences across all behavioral measures, with some participants performing relatively poorly and others performing near ceiling. This is consistent with recent large-scale online studies showing wide variation in navigational ability across individuals and populations (Coutrot et al., 2018, 2022).

While performance was highly consistent between some tasks of scene processing, performance on other tasks was more divergent. Previous analyses of this dataset have already described that overall performance on the Navigation test positively correlates with the SBSOD, spatial SAM, and RM for scenes (Clark et al., 2019; Clark and Maguire, 2020). Similarly, Clark and Maguire (2023) previously reported the high correlation between the SBSOD and spatial SAM, suggesting that both questionnaires measure similar aspects of self-reported spatial cognition. We observed the highest correlation between the overall score and sketch map subscale of the navigation test, due to the sketch map making the largest contribution to the overall score. We also observed high correlations for route knowledge with both the overall score and sketch map subscale. However, proximity judgments between landmarks showed relatively poorer agreement with other scales of the navigation test and with the other measures, indicating this subscale may assess somewhat different aspects of scene perception. Scene perception is a fundamentally multifaceted process. For instance, neural models propose a distinction between cognitive processes underpinning spatial navigation and scene recognition (Dilks et al., 2022). Similarly, spatial navigation has been suggested to utilize representations spanning a continuum between egocentric and allocentric reference frames (Ekstrom et al., 2017). Using multiple distinct behavioral measures therefore allowed assessing multiple aspects of cognitive processing of scenes.

We next measured the resting-state functional connectivity of the core scene regions, including the OPA, pPPA, aPPA, and the RSC. Our analyses replicated previous findings of a posterior–anterior bias within the scene processing network (Baldassano et al., 2013, 2016; Nasr et al., 2013; Silson et al., 2016; Boccia et al., 2017a; Watson and Andrews, 2024). More posterior regions, particularly the OPA, showed preferential connectivity with early visual regions as well as posterior parietal cortices. Meanwhile, more anterior regions, such as the RSC, showed preferential connectivity with the cIPL and hippocampus, and with the frontoparietal control and default mode networks. These biases have previously been taken as suggestive of a role of the posterior scene network in representing egocentric visual features of scenes, which may include low-level visual processes but potentially also higher-level cognitive functions associated with the dorsal visual stream. Conversely, it has been suggested that the anterior scene network may be implicated in higher-level mnemonic processing of scenes.

To directly examine the association between the functional connectivity of the scene network and scene perception, we compared interindividual variability in the strength of these connections to performance on each behavioral measure. Contrary to our predictions, we found no consistent associations between functional connectivity and any of the behavioral measures. We first considered ROI-based analyses, measuring the connectivity of the core scene regions with early visual and extended scene regions or with cortical resting-state networks and subcortical structures throughout the brain. These connections showed only weak associations with the behavioral measures. Although some correlations were significant, very few of these survived a correction for multiple comparisons. More importantly, the effect sizes were very small (with Pearson's *r* values typically <0.2) and included both positive and negative effects. Negative correlations are not readily interpretable, as these imply that decreased resting-state functional connectivity between regions is associated with higher behavioral performance. Furthermore, corresponding Bayes factors indicated support for the null hypothesis in the vast majority of cases. Seed-based analyses revealed a number of clusters showing both positive and negative correlations with behavior. However, these clusters were typically relatively small, were not observed consistently over behavioral measures, and were not consistently located within regions or networks showing preferential connectivity with the scene network.

It may be that human behavior depends on the interaction of multiple connections within and between networks, rather than on the operation of individual connections. To this end, we conducted exploratory analyses of the ROI-based connectivity using ridge regression models that included all connections as predictors simultaneously. This approach allowed integrating predictive information over all connections together. However, these models were also unable to predict performance on the behavioral tasks. Indeed, the cross-validated *R*^2^ values were often negative, indicating even worse performance than a simple mean intercept model. When employing cross-validation, there is no lower limit on the range of possible *R*^2^ values. Thus, while it is clear these regression models performed poorly, it is not unprecedented to obtain negative *R*^2^ values. For instance, Kraljević et al. (2024) report similarly poor performance for ridge regression analyses using resting-state functional connectivity to make out-of-sample predictions of cognitive abilities. Taken together these results demonstrate that interindividual variation in resting-state functional connectivity, either in individual connections or in the interaction between multiple connections, was not predictive of performance on behavioral measures of scene processing.

A potential limitation of our design is that the ROIs were defined at the group-level from an independent localizer dataset. This was necessary as the Clark and Maguire dataset itself does not include a scene localizer task. Future research may benefit from using individualized ROIs that account for interindividual variation in the location of category-selective regions. Nevertheless, despite using group-level ROIs, our analyses successfully replicated previously reported connectivity biases between anterior and posterior regions of the scene network (Baldassano et al., 2016), indicating that our group-level ROIs still adequately captured individual participants' scene-selective regions.

In many cases a null result is not unexpected—we would not predict a correlation between behavior and connections that are not suggested to subserve that behavior (such as connections with primary sensory regions). However, we do not observe positive behavioral associations even with connections that have been posited to underpin higher-level cognitive processes. Previous studies using both resting-state and natural viewing paradigms have reported preferential functional connectivity between scene regions and the dorsal attention, frontoparietal control, and default mode networks (Baldassano et al., 2016; Sulpizio et al., 2016; Boccia et al., 2017a,b, 2019; Tullo et al., 2023; Watson and Andrews, 2024). Although we replicate these results in the current study, we did not observe any consistent associations between these connections and behavioral performance. Note that we failed to replicate the results of Sulpizio et al. (2016), which indicated a positive correlation between navigational ability and resting-state functional connectivity of the right RSC and posterior hippocampus. This may reflect differences in methodology—Sulpizio and colleagues used a median split of behavioral scores (while we treat them as continuous measures) and divided the hippocampus into posterior and anterior subsections (while we considered it as a single region).

The absence of consistent associations between the resting-state functional connectivity of the scene network and behavior raises important questions. One possibility is that functional connections with scene-selective regions do not underlie cognitive processes that are reflected in scene processing abilities. However, such a conclusion would be at odds with the observation that the core scene regions do differentially show preferential connectivity with specific regions and networks, including those implicated in higher-level cognitive processes. An alternative possibility is that such connections do subserve behavior, but our experimental design was insufficient to reveal this. Previous research has demonstrated that brain-wide resting–state functional connectivity is capable of predicting various cognitive abilities, including intelligence, attention, and creative ability (Finn et al., 2015; Rosenberg et al., 2016; Beaty et al., 2018). However, other studies have suggested that resting-state functional connectivity may be a poor predictor of performance on certain cognitive tasks (Marek et al., 2022; Kraljević et al., 2024). The efficacy of resting-state functional connectivity as a predictor may be contingent upon the specific brain networks and behaviors under examination. In the domain of scene perception, there may exist a substantial disconnect between scene processing abilities assessed outside the scanner and the functional connectivity of the scene network measured at rest within the scanner.

To address this issue, an alternative approach would be to measure functional connectivity while participants actively perform tasks relevant to scene processing. For instance, the perception and imagery of familiar places have been shown to modulate the functional connectivity between the PPA, RSC, and hippocampus (Boccia et al., 2017b, 2019; Tullo et al., 2023). A more task-general possibility would be to compare behavior measured outside the scanner with functional connectivity measured during naturalistic viewing, which is intended to provide a more ecologically valid estimate of brain states than resting-state (Finn, 2021). Indeed, movie watching has been shown to outperform resting-state at predicting behavioral traits (Finn and Bandettini, 2021). Furthermore, while our analysis focused exclusively on functional connectivity, future studies could also test associations between behavior and the structural connectivity of the scene network. For example, the microstructure of the fornix (projecting from the hippocampus) has been linked to performance on scene discrimination and navigation tasks (Postans et al., 2014; Hodgetts et al., 2015, 2020).

In conclusion, this study used behavioral and fMRI data from a large public database to compare resting-state functional connectivity of the scene network with performance on behavioral measures of scene perception. We found substantial individual differences in scene recognition, spatial memory, and navigational abilities. Furthermore, there was wide variability in the correlations between behavioral measures, suggesting that the measures assessed different aspects of scene processing. However, we did not find any consistent associations between interindividual variability in functional connectivity and performance on the behavioral measures. Future research is required to clarify the role of these connections in the cognitive processing of scenes, potentially by measuring functional connectivity while engaged in scene-relevant tasks or under more naturalistic viewing conditions.

## Synthesis

Reviewing Editor: Ifat Levy, Yale School of Medicine Decisions are customarily a result of the Reviewing Editor and the peer reviewers coming together and discussing their recommendations until a consensus is reached. When revisions are invited, a fact-based synthesis statement explaining their decision and outlining what is needed to prepare a revision will be listed below. The following reviewer(s) agreed to reveal their identity: Edward Silson.

The authors have adequately addressed most of the reviewers' comments. Please address these remaining comments:

- The authors explain how their analyses and study differ from other successful attempts to link resting state functional connectivity to individual differences in behavior and cognition. However, they don't directly comment on the fact that their R2 values are even worse than predicting the mean. Is this surprising given other work using similar methods?

-The authors say their anterior/posterior PPA ROIs look strange because of the projection from volume to surface. If the visualization method they have chosen does not accurately reflect the ROIs, they should fix this. Please show the ROIs in volumetric MNI space so readers can assess their anatomical location.

## Author Response

The authors have adequately addressed most of the reviewers' comments. Please address these remaining comments:

- The authors explain how their analyses and study differ from other successful attempts to link resting state functional connectivity to individual differences in behavior and cognition. However, they don't directly comment on the fact that their R2 values are even worse than predicting the mean. Is this surprising given other work using similar methods? > When employing cross-validation there is no lower limit on the range of R2 values that can be obtained. Thus, while it is clear our ridge regression models performed poorly, it is not unprecedented to obtain negative R2 values. Indeed, a recent study by Kraljević et al. (2024) also reported negative R2 values when using ridge regression models to make out-of-sample predictions of various cognitive abilities from resting-state functional connectivity. We have updated the Discussion to clarify these points (lines 522-528). As we already discuss, the key issue is that the performance of resting-state functional connectivity in predicting behaviour depends on both the brain networks and behavioural abilities being assessed. In our particular case, resting-state functional connectivity of the scene network was not predictive of behavioural performance on scene processing tasks.

-The authors say their anterior/posterior PPA ROIs look strange because of the projection from volume to surface. If the visualization method they have chosen does not accurately reflect the ROIs, they should fix this. Please show the ROIs in volumetric MNI space so readers can assess their anatomical location. > We chose to visualise the regions of interest on the inflated cortical surface, rather than in the volume, as it is better suited to displaying the arrangement of multiple regions simultaneously across the cortical surface. It also provides a clearer visualisation of V1 and V2. In addition, these plots provide an analogous representation to the surface plots of the seed-based connectivity maps in Figure 4, allowing readers to easily compare between the figures. Nevertheless, we understand that readers may also appreciate a volume-based visualisation of the ROIs. Given the benefits of the surface visualisation mentioned above, we have opted to include a new extended data figure (Figure 1-1) providing an alternative volume-based visualisation, rather than replacing the surface visualisation entirely.
