# Supplementary figures and images for: Functional Connectivity of the Scene Processing Network at Rest Does Not Reliably Predict Human Behavior on Scene Processing Tasks
Source: eNeuro. 2025 Feb 11;12(2):ENEURO.0375-24.2024. doi: 10.1523/ENEURO.0375-24.2024 (PMC11820959; doi:10.1523/ENEURO.0375-24.2024)

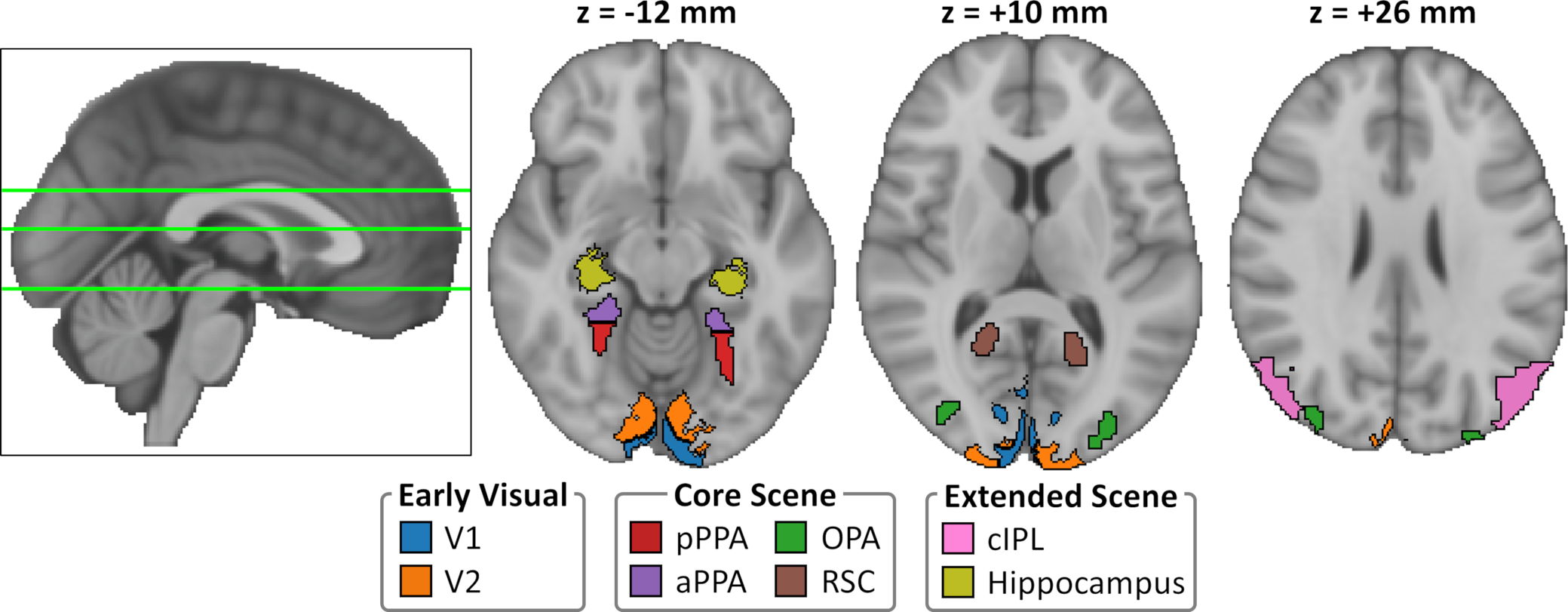

Supplement: Figure 1-1 — Volume-based visualisation of main regions of interest for connectivity analyses: early visual (V1, V2); core scene (Occipital Place Area [OPA], posterior and anterior Parahippocampal Place Area [pPPA, aPPA], Retrosplenial Complex [RSC]); extended scene (caudal Inferior Parietal Lobule [cIPL], Hippocampus). Regions are displayed on the MNI152 brain. Download Figure 1-1, TIF file. [file eneuro-12-ENEURO.0375-24.2024-s002.tif]

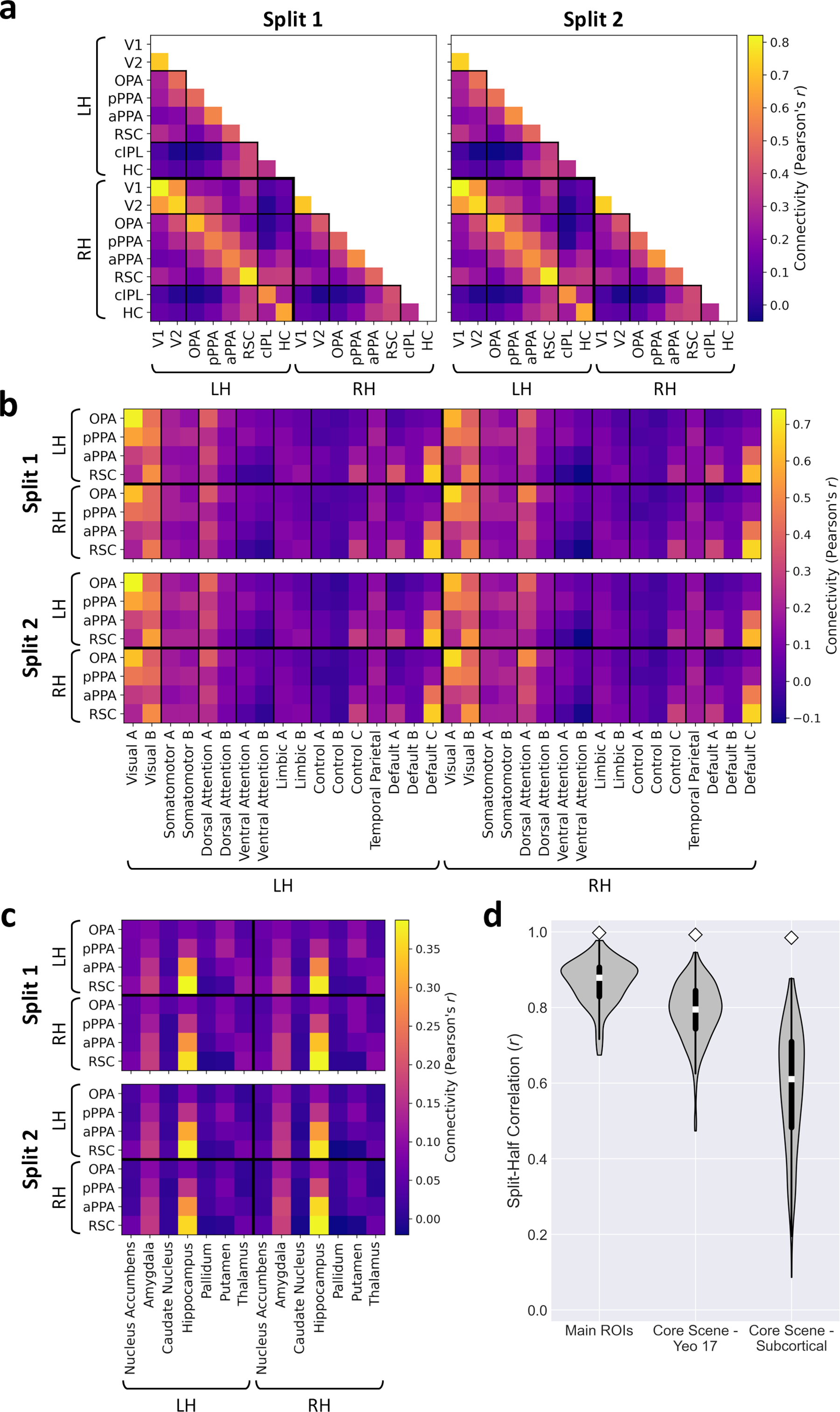

Supplement: Figure 3-1 — Split-half reliability of functional connectivity between first and second halves of resting-state scan runs. Matrices illustrate group average functional connectivity between (a) main regions of interest, (b) core scene regions and 17 cortical resting-state networks, and (c) core scene regions and subcortical regions for each data split. (d) Split-half correlations between connectivity matrices. Violin plots illustrate distributions of correlations over individual subjects. Diamond markers indicate correlations between group average matrices. Download Figure 3-1, TIF file. [file eneuro-12-ENEURO.0375-24.2024-s003.tif]

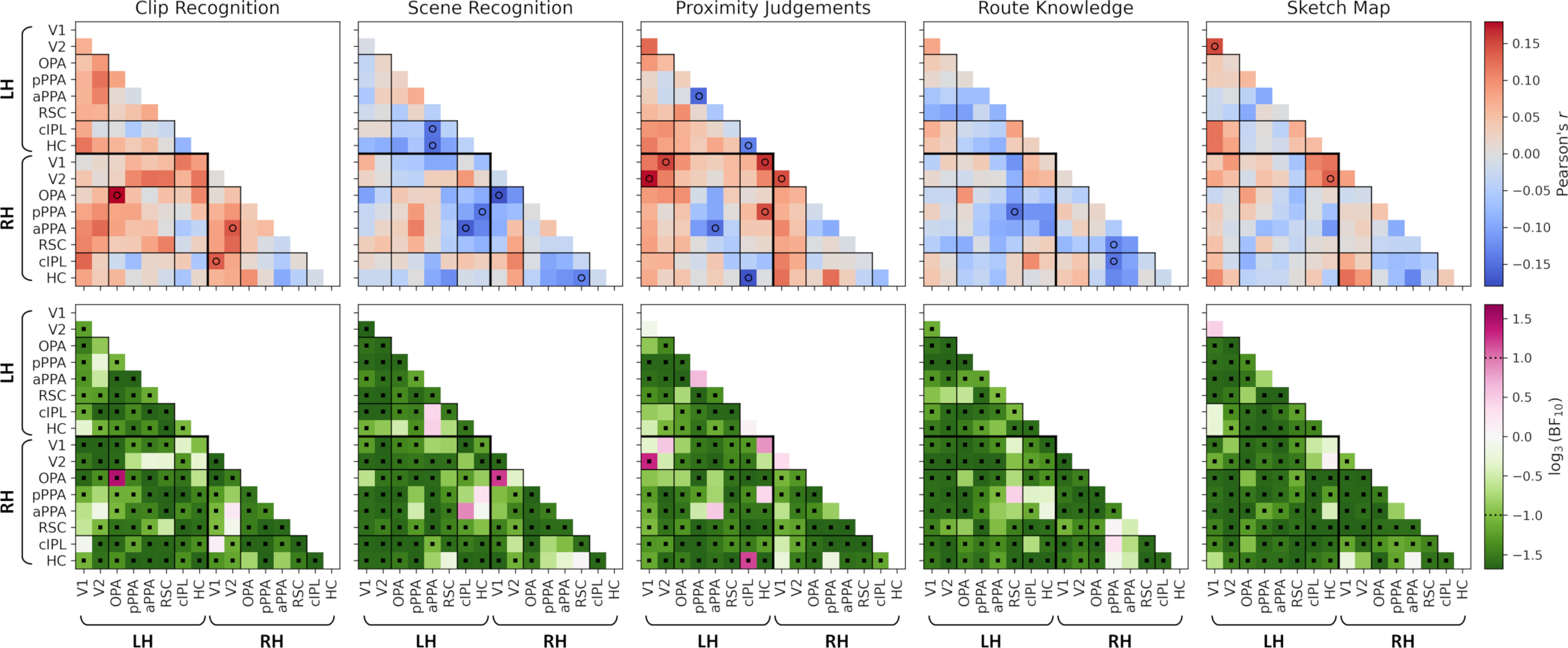

Supplement: Figure 5-1 — Correlations between subscales of the Navigation test and functional connectivity between early visual, core scene, and extended scene regions (cf. Figure 2a). Top row illustrates correlation values – open circles indicate correlations significant at an uncorrected level (p < 0.05). No correlations survived the FWER correction. Bottom row illustrates Bayes factors on a log scale – positive and negative values indicate support for the alternative and null hypotheses respectively. Square markers indicate Bayes factors greater than 3 or less than 1/3. Download Figure 5-1, TIF file. [file eneuro-12-ENEURO.0375-24.2024-s004.tif]

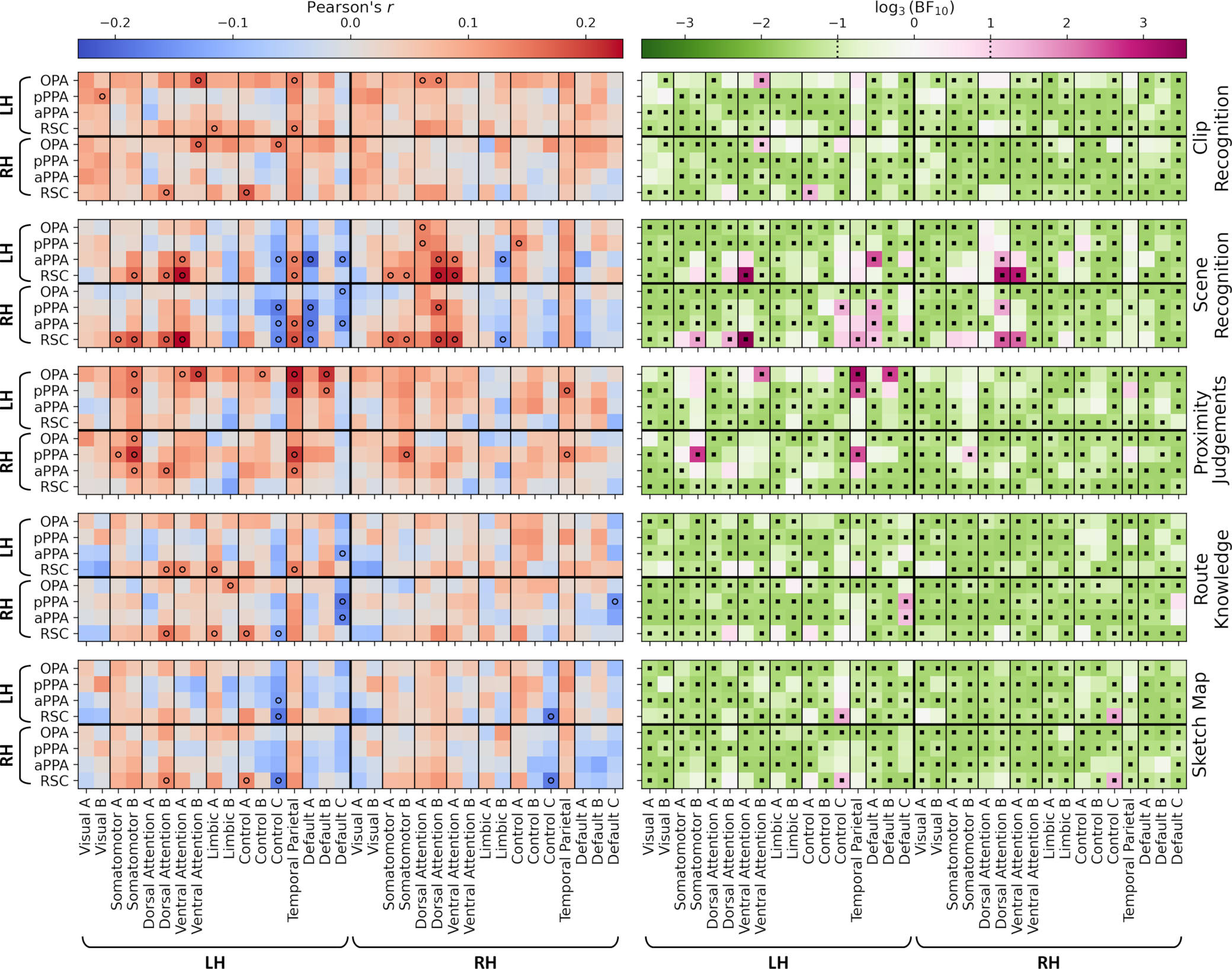

Supplement: Figure 6-1 — Correlations between subscales of the Navigation test and functional connectivity between core scene regions and cortical resting-state networks (cf. Figure 2d). Left column illustrates correlation values – open circles indicate correlations significant at an uncorrected level (p < 0.05). No correlations survived the FWER correction. Right column illustrates Bayes factors on a log scale – positive and negative values indicate support for the alternative and null hypotheses respectively. Square markers indicate Bayes factors greater than 3 or less than 1/3. Download Figure 6-1, TIF file. [file eneuro-12-ENEURO.0375-24.2024-s005.tif]

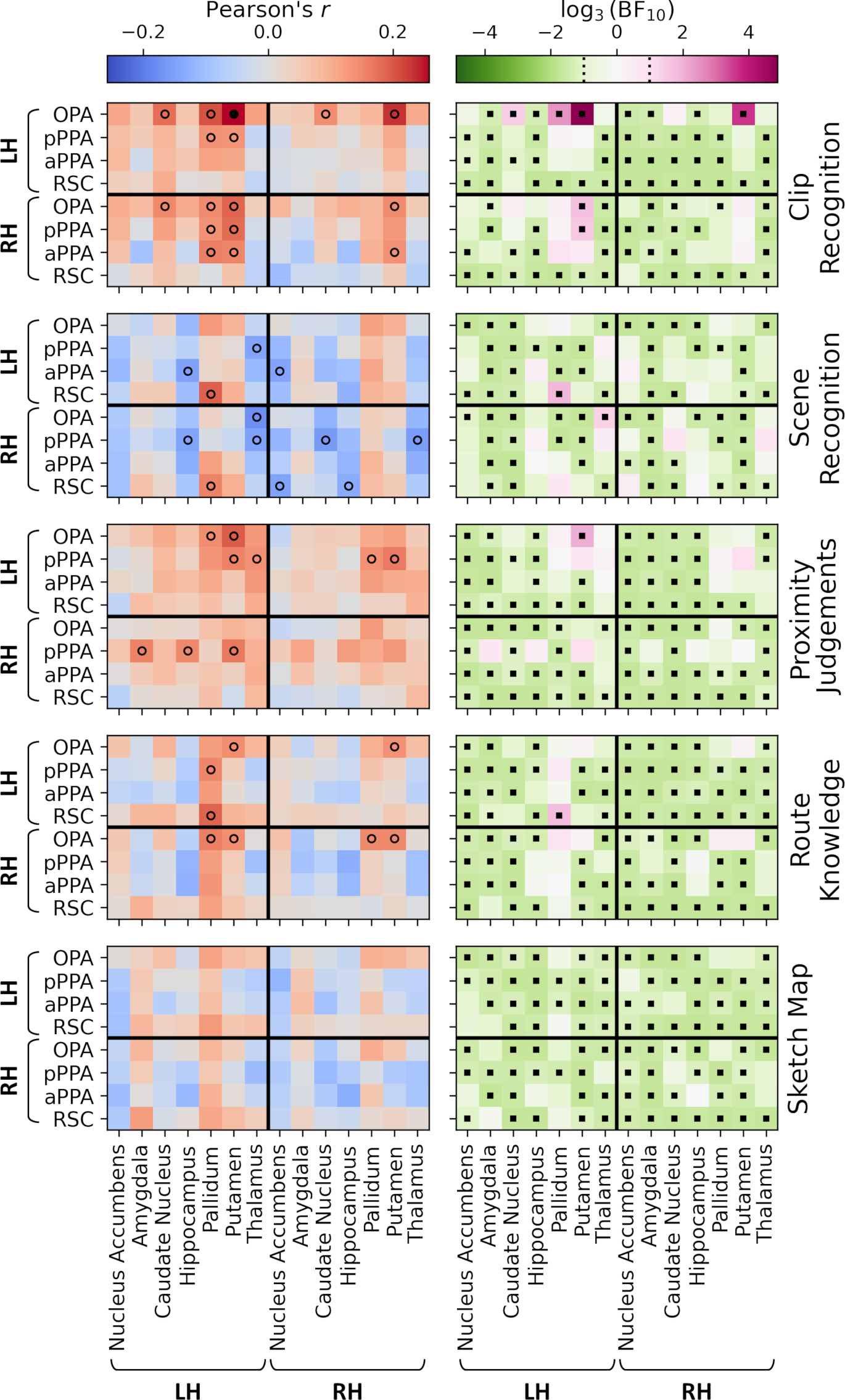

Supplement: Figure 7-1 — Correlations between subscales of the Navigation test and functional connectivity between core scene and subcortical regions (cf. Figure 2e). Left column illustrates correlation values – open circles indicate correlations significant at an uncorrected level; filled circles indicate correlations significant following a FWER correction (p < 0.05). Right column illustrates Bayes factors on a log scale – positive and negative values indicate support for the alternative and null hypotheses respectively. Square markers indicate Bayes factors greater than 3 or less than 1/3. Download Figure 7-1, TIF file. [file eneuro-12-ENEURO.0375-24.2024-s006.tif]
